# Wildlife Tourism Opens Opportunities to Explore Agonistic Interactions Among Elusive Neotropical Cats

**DOI:** 10.1002/ece3.73421

**Published:** 2026-04-14

**Authors:** Fernando R. Tortato, Raíssa Sepulvida, Jorge Barragan, Valeria Boron, Samantha Rincón, Rafael Hoogesteijn

**Affiliations:** ^1^ Panthera Poconé Mato Grosso Brazil; ^2^ Reserva Natural de La Sociedad Civil Hato La Aurora Hato Corozal Colombia; ^3^ World Wide Fund for Nature (WWF), the Living Planet Centre Woking UK; ^4^ Fundação Pró‐Natureza—Funatura Brasília Brazil; ^5^ Wildlife Conservation Society—WCS Paraguay (Consultant Jaguar/Livestock Conflict Resolution) Asunción Paraguay

**Keywords:** agonistic encounters, animal behavior, competition, ecotourism, Felidae

## Abstract

Wildlife tourism is a globally expanding activity that acts as a double‐edged sword, bringing benefits to communities and conservation while potentially affecting species behavior. However, habituation to tourists also serves as a catalyst for behavioral ecology research by allowing the observation of target species. In the Pantanal of Brazil and the Llanos of Colombia, jaguar‐oriented tourism has made possible the recording of unprecedented behaviors. In this paper we present records of agonistic interactions between jaguars and two other wild cats, the puma and the ocelot, involving interspecific killing and intra‐guild predation. We discuss the nature of these interactions and the role of wildlife tourism as a tool to unravel the behaviors of these elusive wild cats of the Americas.

## Graphical abstract

Wildlife tourism in South America offers a unique platform for the noninvasive study of elusive predators such as wild cats. By leveraging data generated from tourist observations, this study reveals new insights into the behavior patterns and ecology of these animals in their natural habitat.

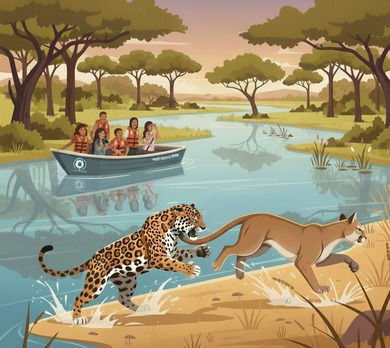

## 1 Introduction and Observation

Wildlife tourism is a globally growing enterprise where tourists seek contact with charismatic species, especially in protected areas (Higginbottom 2004). Estimates indicate that more than 8 billion people per year visit protected areas around the world (Balmford et al. 2015). Among charismatic species, big cat's represent consolidated attractions in wildlife tourism in Africa, Asia and some portions of Latin America (Mossaz et al. 2015; Tortato et al. 2017; Tortato et al. 2020; Ohrens et al. 2021; Hyde et al. 2023; García‐Londoño and Roldán‐Clarà 2025). Wildlife tourism centered on big cat's acts as a double‐edged sword, catalyzing conservation awareness while potentially disrupting ecological and behavioral patterns (Mossaz et al. 2015; Macdonald et al. 2017; Tyagi et al. 2019; Cifuentes‐Ibarra et al. 2023; García‐Londoño and Roldán‐Clarà 2025). While it funds protected areas, poorly managed operations lead to animal stress and culture commodification (Tyagi et al. 2019; García‐Londoño and Roldán‐Clarà 2025; García‐Londoño et al. 2025). Currently, jaguar tourism in the Americas serves as a key conservation tool, yet its sustainability relies on adaptive social strategies (Tortato et al. 2024). Supporting this complexity, studies on pumas suggest that while tourism may not reduce habitat occupancy, it can still trigger subtle behavioral shifts (Cifuentes‐Ibarra et al. 2023). According to recent reviews, primary benefits include habitat protection, improved stakeholder tolerance, and economic incentives for coexistence. Conversely, significant drawbacks involve the disruption of natural hunting strategies and activity budgets, alongside inequitable profit distribution that often excludes local communities and may trigger future social conflicts. Ultimately, maximizing the pros of jaguar and puma tourism requires rigorous management to mitigate its inherent ecological and ethical risks (Tortato and Izzo 2017; Ohrens et al. 2021; García‐Londoño and Roldán‐Clarà 2025; García‐Londoño et al. 2025).

Despite the ecological risks associated with wildlife habituation, this process also serves as a fundamental catalyst for scientific research by facilitating closer observation of elusive species. Recent breakthroughs in the study of puma and jaguar ethology have predominantly occurred in areas where tourism has fostered a high degree of tolerance to human presence. This habituation significantly increases the probability of researchers, tourists, and local stakeholders documenting rare behaviors through high‐quality media. In Chile, novel behaviors have been described for the puma, such as the first recorded consumption of lesser rhea eggs (Cardenas et al. 2021) and female–female mounting (Lagos et al. 2020). For jaguars, there is an advance in knowledge of behaviors such as female counterstrategies to infanticide (Stasiukynas et al. 2021), coalition of males (Jędrzejewski et al. 2022), and antagonistic interactions with giant otters ( *Pteronura brasiliensis* ) (Leuchtenberger et al. 2016, 2025), with white‐lipped peccaries ( *Tayassu pecari* ) (Rampim et al. 2020), and with maned wolves (Fragoso et al. 2021).

As an apex predator with opportunistic habits, the jaguar's diet includes more than 85 vertebrate species, spanning various mammals, reptiles, fish, and birds (Seymour 1989; González and Miller 2002). There are records of jaguar predation in all Neotropical carnivorous families (Seymour 1989; Weckel et al. 2006). Among the felids, there are records of predation on ocelots ( *Leopardus pardalis* ) (Oliveira and Pereira 2013; Perera‐Romero et al. 2020) and pumas ( *Puma concolor* ) (Crawshaw Jr. and Quigley 2002; Oliveira and Pereira 2013). Jaguar predation on other felids can be considered as intra‐guild predation (IP) when there is killing and eating behavior among potential competitors and interspecific killing (IK) when there is just killing behavior (Polis et al. 1989; Oliveira and Pereira 2013). Due to the species' cryptic nature, IP is typically detected through fecal analysis (e.g., Weckel et al. 2006; Gonzalez‐Maya et al. 2010), while IK events are rarely documented, usually requiring opportunistic camera‐trap footage or direct observation (Crawshaw Jr. and Quigley 2002; Perera‐Romero et al. 2020) and interspecific killing appears common in mammalian carnivores, accounting for 38% of known mortalities; however, there is little information on the ecological and behavioral factors related to these events (Palomares and Caro 1999). Due to the risk of these agonistic interactions, the presence of the jaguar can influence the spatial structure of mesocarnivore population, such as crab‐eating raccoons ( *Procyon cancrivorus* ), ocelots, jaguarundis ( *Puma yagouaroundi* ) and tayras ( *Eira barbara* ) (Boron et al. 2023).

The Pantanal is recognized for having significant populations of jaguars (Soisalo and Cavalcanti 2006; Tomas et al. 2019), pumas (Azevedo et al. 2013), and ocelots (Trolle and Kéry 2003; Tortato et al. 2021). The Colombian Llanos also have important populations of jaguars (Boron et al. 2016; Hyde et al. 2023), pumas, and ocelots (Boron et al. 2021). Both regions share a landscape dominated by native grasslands dissected by riparian forests, where extensive cattle ranching is the primary land use (IDEAM 2014; Tomas et al. 2019). However, these areas have recently emerged as premier ecotourism destinations, with several ranches transitioning into wildlife‐focused enterprises that facilitate the direct observation of jaguars and pumas (Hoogesteijn et al. 2016; Tortato et al. 2017). Due to the high visibility afforded by these open environments and the habituation of the animals, both the Pantanal and the Llanos provide unique opportunities to document the behaviors and interspecific interactions of otherwise cryptic species. In this study, we describe four agonistic interactions between jaguars, pumas, and ocelots documented in tourism hotspots within the Brazilian Pantanal and the Colombian Llanos.

### 1.1 Jaguar Attacking and Chasing Puma

**FIGURE 1:**
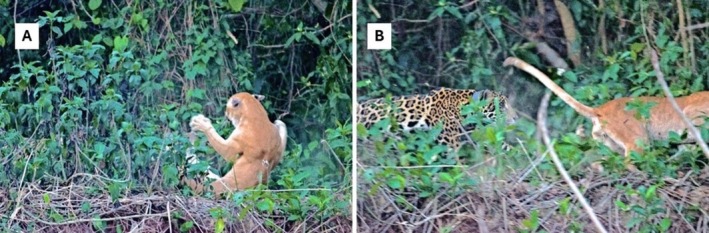
(A) An agonistic encounter between a jaguar ( *Panthera onca* ) and a puma (Puma conccolor) on the banks of the Cuiabá River, witnessed by a group of tourists. (B) The puma fleeing from the jaguar. Credit: Rudolf Schnorrer.

On October 15, 2011, a group of German tourists guided by Adilson Draeger observed a female jaguar resting on the banks of the Piquiri River, Corumbá, Mato Grosso do Sul, Brazil. The female jaguar saw a puma approaching in the same riverbank and immediately attacked it and chased it, making the puma run away (Figure 1). After the agonistic interaction, the jaguar returned to the riverbank and came back to rest. According to the tourists, the agonistic interaction lasted less than 2 min. Through the photos kindly provided by Rudolf Schnorrer, it is possible to see that the jaguar and puma have similar sizes. The jaguar was a female monitored by GPS collar by Panthera and at the time of capture weighed 63 kg.

### 1.2 Intra‐Guild Predation Between Jaguar and Ocelot

On April 30, 2014, during the night (08:00 pm), RHR observed an adult male jaguar consuming prey near the Cuiabá River and the headquarters of Fazenda São Bento (17°19′35.51″S; 56°44′18.41″O), Corumbá, Mato Grosso do Sul, Brazil. This event took place on a cattle ranch and research base adjacent to the tourist area, and the jaguar observed was an animal habituated to human presence. With the use of a flashlight, it was possible to observe an adult male jaguar, but it was not possible to identify the consumed prey. On the following morning, RHR returned to the event site and found the carcass of an ocelot totally consumed.

### 1.3 Interspecific Killing Between Jaguar and Ocelot

**FIGURE 2:**
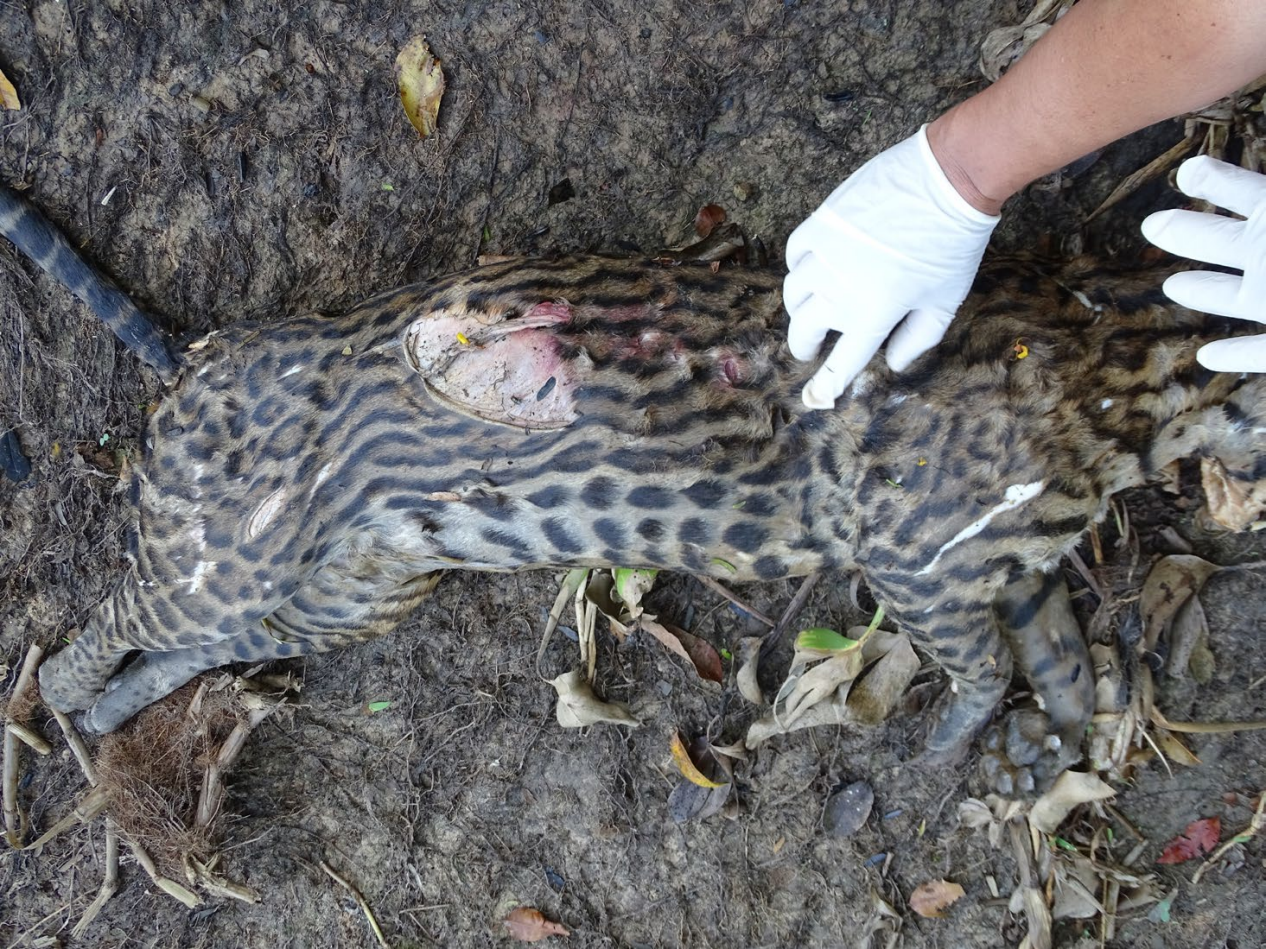
Adult ocelot ( *Leopardus pardalis* ) killed by a jaguar at Encontro das Águas State Park. Bite and scratch marks indicate an agonistic encounter; the carcass was not consumed.

On September 2, 2014, a killed adult male ocelot was found by tourists on the banks of the Corixo Negro river, Encontro das Águas State Park (17°16′34.74″S; 56°41′25.38″O), Barão de Melgaço, Mato Grosso, Brazil. A biopsy was performed, identifying a bite on the back with > 7 cm between canines, consistent with the attack of a jaguar. There were also scratch marks, indicating the agonistic interaction between the two felines (Figure 2). The ocelot was killed but not consumed.

### 1.4 Intra‐Guild Predation Between Jaguar and Puma

**FIGURE 3:**
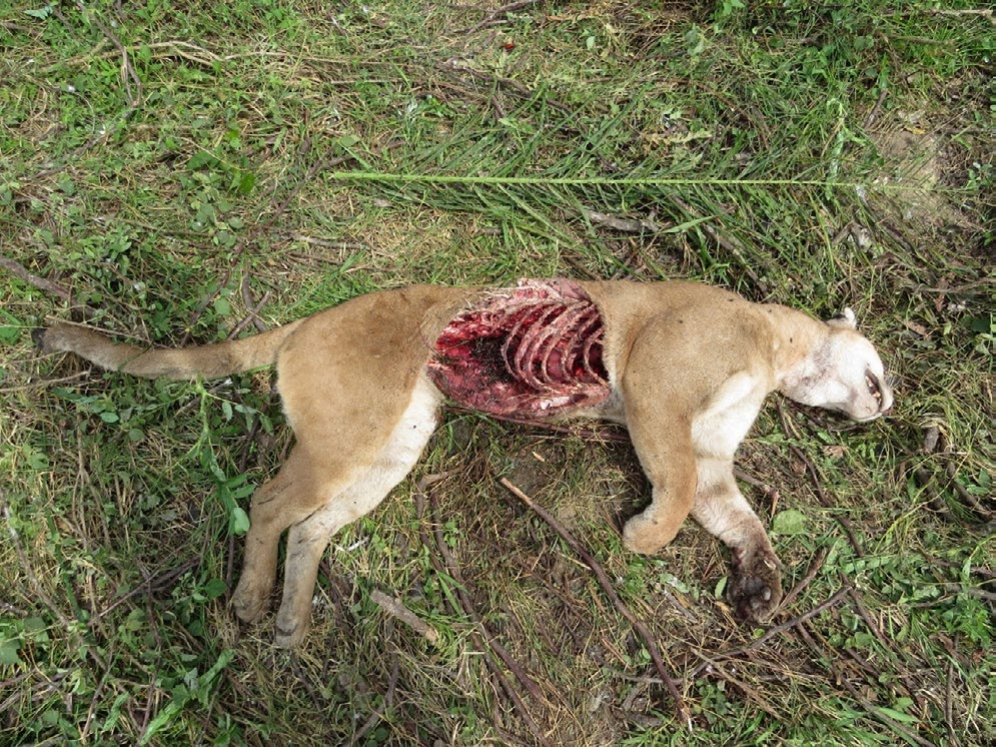
Adult puma killed and partially consumed by a jaguar in the Llanos of Casanare, Colombia. Deadly cranium wounds and missing claws indicate a violent territorial struggle.

On December 31st, 2016 a puma was found killed in the Chamuscada sector of Hato La Aurora (6°0′39.888″N; 71°24′16.200″W), in the Llanos of Casanare, Colombia. This property develops big cat observation tourism, and during routine tourism activities, this case was documented. The Chamuscada sector was part of the territory of two adult jaguar males. The puma's body was found with multiple deadly wounds in the cranium, cuts in the paws, and missing claws, which all suggest a fight endured with a jaguar and consequent death. Its right flank was eaten (Figure 3), while the right side of the skull and the orbital cavities were destroyed. It was an adult puma, weighing 65–70 kg and measuring 1.98 m from its nose to the tip of the tail.

## 2 Discussion

Dominance behavior is determined by body size (Oliveira and Pereira 2013; Palomares and Caro 1999). For example, ocelots exert great killing pressure in many carnivore species (Oliveira and Pereira 2013), the “ocelot effect” (Oliveira et al. 2010), with great impact on smaller sized felids. Events of interspecific killing between jaguars and pumas and jaguars and ocelots are likely to occur given their differential body size (Oliveira and Pereira 2013), but they are rarely recorded. If we consider the two events reported here, there are now six published cases of interspecific killing and predation between jaguars and ocelots (Chinchilla 1997; Gonzalez‐Maya et al. 2010; Oliveira and Pereira 2013; Perera‐Romero et al. 2020), and four published cases in scientific papers for jaguars and pumas (Crawshaw and Quigley 1984; Crawshaw Jr. and Quigley 2002; Harmsen et al. 2009; Ruth and Murphy 2010; Elbroch and Kusler 2018). The interactions reported in this study are distinct by the fact that in some events there was the killing and consumption of the ocelot and the puma (IP); in another event, it was only the killing of an ocelot, without any energetic gain (IK); and in the remaining event it was an attack of a jaguar on a puma without leading to death. The nonconsumption may be due to the availability of alternative prey (Palomares and Caro 1999) or to the competitive nature of the killing (Oliveira and Pereira 2013). Oliveira and Pereira (2013) reviewed the interactions between carnivores in South America and concluded that they are not random and play an important role in structuring carnivore communities, either by affecting population density, space use or activity pattern (Palomares and Caro 1999; Lewis et al. 2015).

Ocelot, puma and jaguar distributions significantly overlap, from the southern United States to Argentina (Hunter 2011). The preference for forested habitats by jaguars (Morato et al. 2018) and ocelots (Bianchi et al. 2016) and an overlapping activity pattern of jaguars, pumas and ocelots (Santos et al. 2019; Perera‐Romero et al. 2020), enhance the interactions between these wild cats in the Americas. These interactions can be even more frequent in the Pantanal and Llanos, since the forested areas present high cat densities, surrounded by vast open and humid areas (Devlin et al. 2023; Hyde et al. 2023). In these well‐preserved landscapes, which remain free from significant anthropogenic pressures, the high spatial overlap and subsequent encounters are primarily driven by the natural productivity and the ecological structure of these areas. These cases reported here were located near forested areas and wetland areas. Water resources, especially during dry seasons, promotes multiple predator—prey and predator–predator interactions. Perera‐Romero et al. (2020) reported an event of ocelot predation by jaguar in a water source in Guatemala. The data presented here reinforce that the IP and IK caused by the jaguar can be a relevant factor to shape the distribution and abundance of ocelots and pumas. The nontolerance behavior of the jaguar towards the puma may also shape the distribution of the puma.

Jaguar tourism offers a unique opportunity to observe and document elusive species, especially in landscapes like the Pantanal and Llanos, where high visibility and established research partnerships facilitate unprecedented data collection. This growth in jaguar‐focused enterprises has shifted local attitudes from conflict to coexistence (Harris et al. 2005; Tortato et al. 2017), fostering a degree of habituation that acts as a fundamental catalyst for behavioral ecology research. While such proximity increases the chances of documenting rare antagonist interactions like the ones reported here, this behavioral desensitization carries inherent conservation risks. For instance, reduced fear may lead large felids to frequent human settlements or livestock areas more often, potentially increasing the risk of human‐wildlife conflict on properties adjacent to tourist sites (Ohrens et al. 2021; García‐Londoño and Roldán‐Clarà 2025). Ultimately, as discussed in our initial considerations, while habituation involves these ecological risks, it remains a valuable tool for science. Wildlife tourism development in these areas can, therefore, serve as a strategic partner to both jaguar conservation and behavioral ecology by deepening our understanding of how such interactions impact sympatric species populations.

## Author Contributions

**Fernando R. Tortato:** conceptualization (lead), investigation (equal), methodology (equal), supervision (equal), writing – original draft (lead), writing – review and editing (equal). **Raíssa Sepulvida:** investigation (equal), methodology (equal), writing – review and editing (equal). **Jorge Barragan:** investigation (equal), methodology (equal), writing – review and editing (equal). **Valeria Boron:** conceptualization (equal), investigation (equal), methodology (equal), writing – review and editing (equal). **Samantha Rincón:** investigation (equal), methodology (equal), writing – review and editing (equal). **Rafael Hoogesteijn:** conceptualization (equal), investigation (equal), methodology (equal), writing – review and editing (equal).

## Funding

This work was supported by Panthera, which provided both financial and logistical support at its research station in the Brazilian Pantanal.

## Conflicts of Interest

The authors declare no conflicts of interest.

## Data Availability

The data that support the findings of this study are available within the article.
